# Rationale and protocol of the StayFitLonger study: a multicentre trial to measure efficacy and adherence of a home-based computerised multidomain intervention in healthy older adults

**DOI:** 10.1186/s12877-020-01709-2

**Published:** 2020-08-28

**Authors:** S. Belleville, M. Cuesta, M. Bieler-Aeschlimann, K. Giacomino, A. Widmer, A. G. Mittaz Hager, D. Perez-Marcos, S. Cardin, B. Boller, N. Bier, M. Aubertin-Leheudre, L. Bherer, N. Berryman, S. Agrigoroaei, J. F. Demonet

**Affiliations:** 1 Research Centre, Institut universitaire de gériatrie de Montréal, CIUSSS du Centre-Sud-de-l’Île-de-Montréal, 4565, Chemin Queen-Mary, Montréal, Québec H3W 1W5 Canada; 2 Université de Montréal, Montréal, Canada; 3 Leenaards Memory Centre, University Hospital of Lausanne, Lausanne, Switzerland; 4 MindMaze SA, Lausanne, Switzerland; 5 HES-SO Valais-Wallis, School of Health Sciences, Loèche-les-Bains, Switzerland; 6 HES-SO Valais-Wallis, School of Managment, Sierre, Switzerland; 7 Université du Québec à Trois-Rivières, Trois-Rivières, Canada; 8 Université du Québec à Montréal, Montréal, Canada; 9 Montréal Heart Institute, Montréal, Canada; 10 Psychological Sciences Research Institute, Université catholique de Louvain, Louvain-la-Neuve, Belgium

**Keywords:** Cognitive training, Physical activity training, Social interactions, Home-based training, Computerised training, Multidomain intervention, Adherence, Frailty, Cognition

## Abstract

**Background:**

In older adults, multidomain training that includes physical and cognitive activities has been associated with improvement of physical and cognitive health. The goal of the multisite StayFitLonger study is to assess a home-based computerised training programme, which combines physical exercises, stimulating cognitive activities and virtual coaching.

**Methods:**

One hundred twenty-eight cognitively healthy older adults will be recruited from the community in Switzerland, Canada and Belgium. The study will comprise (1) a 26-week double-blind randomized controlled efficacy trial and (2) a 22-week pragmatic adherence sub-study. In the efficacy trial, participants will be randomly assigned to an experimental or an active control intervention. In the experimental intervention, participants will use the StayFitLonger programme, which is computerised on a tablet and provides content that combines physical activities with a focus on strength and balance, as well as divided attention, problem solving and memory training. Outcomes will be measured before and after 26 weeks of training. The primary efficacy outcome will be performance on the “Timed-Up & Go” test. Secondary outcomes will include measures of frailty, cognition, mood, fear of falling, quality of life, and activities of daily living. Age, sex, education, baseline cognition, expectation, and adherence will be used as moderators of efficacy. Following the 26-week efficacy trial, all participants will use the experimental programme meaning that participants in the control group will ‘cross over’ to receive the StayFitLonger programme for 22 weeks. Adherence will be measured in both groups based on dose, volume and frequency of use. In addition, participants’ perception of the programme and its functionalities will be characterised through usability, acceptability and user experience.

**Discussion:**

This study will determine the efficacy, adherence and participants’ perception of a home-based multidomain intervention programme and its functionalities. This will allow for further development and possible commercialization of a scientifically validated training programme.

**Trial registration:**

ClinicalTrials.gov, NCT04237519 Registered on January 22, 2020 - Retrospectively registered.

## Background

Finding ways to improve and maintain functional abilities and quality of life in older adults has become a worldwide priority. It is well recognized that a reduced engagement in physical, cognitive and social activities has a negative influence on the health of older adults, exposing them to being more vulnerable both physically and cognitively. Sedentary behaviours can ultimately lead to physical frailty, which is defined as a state of high vulnerability with cumulation of adverse health outcomes [1, 2]. Fear of falling and/or unsteady gait is a common component of physical frailty and falls are particularly frequent in older adults [3, 4]. In addition to mobility limitation and falls, cognitive decline has been identified as a major cause of disability and dependency in older populations [5, 6].

Expert recommendations propose that non-pharmacological interventions focusing on modifiable lifestyle factors can be used to protect older people from the deleterious effects of physical and brain aging that can lead to disability [7, 8]. Keeping a healthy mind in a healthy body might be the approach of choice for healthy aging. Several studies have shown that physical activity induces many beneficial effects on general health, cognition and quality of life in healthy older adults but also in frail individuals [9–14]. In parallel to studies on physical activity, increasing evidence shows that cognitive training can also have a positive impact not only on cognition but also on physical status [15–23]. This is consistent with findings indicating that cognitive deficits, mainly impairment of executive functions and attentional control, are associated with falls [24] and abnormal gait [25].

Because aging is complex and different interventions are likely to potentiate their effects, an increasing number of studies have relied on combined interventions targeting two or more modifiable factors (for a review see [26]). For instance, the FINGER study, which combined face-to-face physical exercises and diet guidance with a home-based computerised-cognitive training, showed cognitive improvement on processing speed and executive functions [27]. The MAPT study used a multidomain intervention, which combined face-to-face cognitive training, diet and physical exercises guidance [28]. However, as these programmes were provided face-to-face for the most part, accessibility remains a potential barrier, as older adults may experience mobility challenges or may not have easy access to resources or facilities that can provide those programmes in their community or nearby environment.

Relying on computerisation to deliver lifestyle interventions has several advantages: it can be used to support home-based training, which reduces costs and increases access; training can be self-paced and repeated as wished; it helps provide immediate feedback; it allows scaling up for wider use if efficacy is proven; and it provides an excellent interface for active control interventions [29–31]. Surprisingly, whereas many studies assessed computerised cognitive training programmes, very few have combined at-home computerised cognitive and physical activity training [32–35]. Furthermore, few studies have integrated and assessed the user viewpoint. Adoption of technology by older adults depends on whether it responds to their needs and whether it is adapted to their capabilities [36, 37]. Barriers of technical nature (e.g., difficulty logging in or navigating) are often raised by older adults when measuring their interest for computerised brain health programmes [38]. This stresses the importance of collecting data on the perception of the programme and its functionalities and working with developers to adapt programmes to end users. This will be done in the present study by measuring usability, acceptability and user experience.

The StayFitLonger study was designed to test efficacy, adherence and perception of a home-based computerised training programme, which combines physical exercises and cognitive training in both robust and pre-frail older adults. The ultimate goal of the training programme is to maintain independent living at home by upholding and when possible improving physical and cognitive capacities in older adults. The programme comprises easily implemented videos of physical exercises focusing on gait and strength (Test-and-Exercise home-based programme, T&E, [39]). It also includes a series of ludic activities to increase cognitive functions. These cognitive activities train attentional control through dual-task exercises that were found to increase divided attention capacities and frontal lobe function [40], general knowledge learning and problem-solving capacities. Other features of the programme include: 1) prospective memory exercises embedded in the physical exercises; 2) social functionalities (i.e., creating and sharing learning material with peers; chatting with peers about topics of interest and sharing solutions to common real-life problems) to encourage social engagement, as it is positively related to health status and cognitive functions in older adults and helps counteract isolation [41, 42]; 3) psychoeducational content on cognition, physical health, nutrition and on ways to apply newly learned strategies in real life to empower participants and promote self-management (e.g., [43]); 4) a virtual coach to improve adherence by guiding participants, reminding them to use the programme regularly, and providing feedback and rewards through a system of virtual credits; 5) possibility to personalise the application settings to tailor the environment to the participant’s tastes and wishes (e.g. virtual coach apparence); and 6) wearable motion sensors, which are used during the physical exercises and one cognitive exercise in which a motor response is required, and as a complement to secondary outcomes.

## Objectives and hypotheses

The StayFitLonger study has two major objectives that will be addressed in the trial and the sub study. The **efficacy trial** will test the effect of the training on physical, cognitive, affective, and psychosocial outcomes using a 26-week double-blind parallel-group randomised control trial (RCT). Participants will be allocated randomly to either the StayFitLonger training home-based computerised programme (experimental intervention) or a home-based computerised comparator (active control). The primary objective is to assess whether the StayFitLonger programme leads to larger pre-post improvement than the active control condition on the Timed-Up & Go (TUG), a broadly used and validated functional physical task to measure lower extremity function, mobility and balance. Participants allocated to the experimental intervention are expected to show larger post-training improvement on the TUG than participants in the control intervention. As a secondary objective, we will assess whether the StayFitLonger programme improves physical, cognitive, affective, and psychosocial secondary outcomes. We will also explore whether gains differs in robust vs. pre-frail seniors since some studies suggest that changes in response to training might depend on frailty status [11, 14].

The **adherence sub-study** will rely on a pragmatic quasi-experimental design. At the end of the 26-week RCT, participants in the experimental group will be asked to continue using the programme and participants in the control group will ‘cross-over’ to the StayFitLonger programme. This sub-study will last 22 weeks and indicators of adherence will be recorded throughout the entire duration of the StayFitLonger study (48 weeks). This will allow us to assess whether adherence is maintained over time and whether it is influenced by personal characteristics, the presence or not of supervision and the type of intervention. Usability, acceptability and user experience will also be evaluated.

## Methods

The study is registered with the US National Institutes of Health clinical trials registry (ClinicalTrials.gov Identifier: NCT04237519). This trial report follows the recommendations of SPIRIT 2013 [44].

### Efficacy trial

#### Design

The design of the efficacy trial and adherence sub-study is illustrated in Table 1. The efficacy trial will be a double-blind parallel group multicentric RCT. It will be completed in three sites: Centre Leenaards de la mémoire – Centre hospitalier universitaire Vaudois (CHUV) in Switzerland; Institut universitaire de gériatrie de Montréal (IUGM) of the Centre intégré universitaire de santé et de services sociaux Centre-Sud-de-l’Île-de-Montréal (CIUSSS-CSMTL) in Canada; and Brusano and Centre Public d’Action Sociale (CPAS) of Woluwe-Saint-Lambert in Belgium. Participants will be randomised to one of two home-based computerised intervention conditions, the StayFitLonger training programme (experimental) or the comparator, an active control training programme. Outcome measures (Table 2) will be collected at two timepoints: pre-training (T0; within 6 weeks prior to the start of the intervention) and post-training (T1, within 4 weeks following the end of the 26-week training). Of note, a second exploratory post-training assessment (T2), not part of the RCT, will be performed within 4 weeks following the end of the adherence sub-study. At each timepoint, there will be two assessment visits. Within a month following the T0 assessment, introductory courses in groups of a maximum of six people will take place to introduce the features of the programme and describe the different physical and cognitive exercises. This will mark the beginning of the training that will take place at home for 26 weeks (see Table 1 for details). Participants will be supervised through home visits and monthly phone calls to monitor their use in relation to recommendation and address problems with the use of the programme.

**Table 1:**
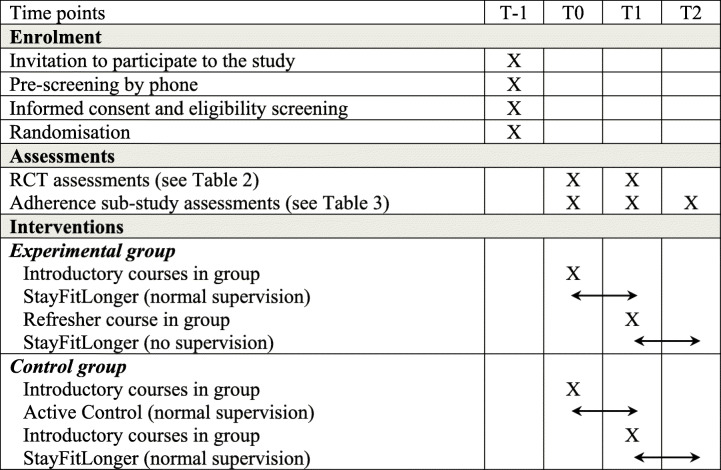
Schedule of enrolment, assessments and interventions

**Table 2:** List of outcomes measured for the RCT

RCT Outcomes				Timepoints		
***Domain***	***Primary outcome***			T0	T1	T2^a^
Physical	Mobility/gait	Timed-Up & Go (TUG) task		X	X	X
***Domain***	***Secondary outcomes***					
Physical	Mobility/balance	20-m Walking task		X	X	X
		Five Time Sit to Stand Test		X	X	X
		Four Stage Balance Test		X	X	X
		Gait Up sensor measurements: several walking parameters		X	X	X
Cognitive	Global cognition: ZAVEN Composite Score	Episodic memory composite	California Verbal Learning Test (CVLT): free delayed recall	X	X	X
			Wechsler Memory Scale (WMS-IV) Logical Memory Test: delayed recall	X	X	X
		Complex attention composite	Wechsler Adult Intelligence Scale (WAIS-IV) Digit Symbol Substitution Test (DSST)	X	X	X
		Executive function composite	Verbal Fluency task	X	X	X
	Memory Composite Score	CVLT: free delayed recall		X	X	X
		WMS-IV Logical Memory Test: delayed recall		X	X	X
	Executive and attentional functions Composite Score	Verbal Fluency test		X	X	X
		Trail Making Test: Part B- Part A		X	X	X
		Victoria Stroop Test (VST): high interference		X	X	X
		Test of Attention Performance (TAP): Divided Attention		X	X	X
	Speed processing Composite Score	Trail Making Test: Part A		X	X	X
		WAIS-IV DSST		X	X	X
		VST: “naming” condition		X	X	X
	Divided attention	Ad-hoc computerised multitasking task		X	X	X
	Prospective memory	Prospective memory items of the Rivermead Behavioural Memory Test (RBMT-3)		X	X	X
	Concept elaboration	Test of Attention Performance (TAP): Flexibility		X	X	X
		WAIS-IV Similarities		X	X	X
Affective	Mood	Hospital Anxiety and Depression Scale (HADS)		X	X	X
	Fear of falling	Falls Efficacy Scale International (FES-I)		X	X	X
Psycho-social	Quality of Life	The Older People Quality of Life questionnaire (OPQOL 35)		X	X	X
	Subjective difficulties encountered in activities of daily living	Cognitive Function Instrument (CFI)		X	X	X
		Everyday Cognition (E-Cog)		X	X	X
	Expectation questionnaire	Ad-hoc questionnaire on participant’s expectation on the programme		X	X	X

^a^T2 assessment is listed here but it is not technically part of the RCT

#### Study population

One hundred and twenty-eight French-speaking healthy participants will be recruited, 64 in Switzerland, 32 in Canada and 32 in Belgium. Participants will be community-dwelling older adults. They will be recruited through diverse sources including ads, newsletters, social media, and flyer distribution during various events. Recruitment will be carried out with the help of two community associations, Pro Senectute in Switzerland and Brusano in Belgium, and from the bank of participants of the IUGM research centre in Canada.

##### Inclusion criteria

Included participants will be fluent French-speaking adults aged 60 years and over, retired and living at home. They will have access to a wireless Internet connection at home and will be open to the use of new technologies including electronic tablets. They will be independent for daily activities based on a normal score on the 4-Instrumental Activities of Daily Living (4-IADL) scale [45]. They will be interested in exercising to stay fit and able to walk at home without a walking aid (e.g., wheelchair, cane, walker, etc.). They will be available to commit themselves for the time period during which the study will take place, with no vision deficits that would prevent them to read information on a tablet and with no current neurological or psychiatric diagnosis (e.g., Parkinson’s disease).

##### Exclusion criteria

Participants with a MoCA score < 26 [46] or a score ≥ 3 on the Fried’s frailty index [2] will be excluded from the study.

#### Procedure and data management

A two-stage screening process will be used to select participants (timepoint T-1; Table 1). Initial contact will be made by phone by research team members with expertise in recruitment. At this stage, only participants who report no major physical, medical, or sensory limitations will be invited to come to the laboratory for further investigation. During the on-site visit, participants will be presented with the information and consent form. In Switzerland, participants will be offered to receive the information and consent form prior to their visit. Once they sign the consent form, inclusion and exclusion entry criteria will be measured for the second-stage screening. The Fried’s phenotype scale [2] will be used to exclude frail individuals and to determine frailty level among other participants who will be identified as either robust (score of 0) or pre-frail (score of 1 or 2). In addition, the participant’s technology (e.g., use of tablet, email, social network) and gaming profile will be established with an ad-hoc questionnaire. Eligible participants will receive an ID number. All data will be anonymized and maintained in REDCap, a secure online database [47]. Access to data will be restricted by type of data (e.g., assessors will only have access to assessment data). Furthermore, data collected directly by the programme will be transmitted and maintained in a secured server located at the Haute École Spécialisée de Suisse Occidentale (HES-SO).

#### Randomisation and blinding procedure

A randomisation list will be generated in Switzerland, independently from the research project and implemented using REDCap. In each site, a team member not involved in assessment or monitoring will assign participants by pressing a “randomisation” button on REDCap. A stratification will be done according to the frailty status. Within each stratum (robust and pre-frail), participants will be assigned to one of the two conditions (StayFitLonger or active control) according to separate randomisation schedules with a 1:1 ratio. Couples (e.g., married individuals) who participate in the study will be assigned to one of the two conditions as a pair: the first member of the couple will be randomised, and the second will be assigned to the same intervention. This has been implemented to avoid contamination in cases where two individuals living in the same household would be randomized to different training programmes.

Assessors will be blind to the hypotheses and to participants’ assignment, as they will only have access to the testing sessions. Participants will be asked not to discuss their training programme with assessors. If such a circumstance were to occur, it will be reported but should have minimal effect on data integrity, as assessors will be blinded to the hypotheses. Research team members responsible for the statistical analyses will be blind to the training conditions. Study coordinators and instructors involved in the introductory courses and supervision of home-based training will not be blind. Participants will be aware that the trial has two different training conditions and that they will be randomly allocated to one of them. However, they will not be informed of the study hypotheses and therefore will not know which one is the experimental condition. Both programmes will have a similar main screen layout and name, and the wording of the recruitment documents and consent forms will not convey the notion that one condition is hypothesised as inferior in terms of its effects on physical capacities and cognition [48].

#### Interventions

##### Introductory courses

Four face-to-face introductory sessions will be provided to familiarise participants with the material and the assigned application (Table 1). Two sessions of 3 h will present how to use the tablet and accessories (e.g., handling, charging), how to navigate in the application and how to complete the cognitive exercises. Two sessions of 2 h will present the physical exercises and teach participants how to place the motion sensor (Physilog®5, GaitUp, Switzerland) that will be used to record bodily measurements. In both programmes, physical activity instructors will ask participants to practice physical exercises for a total of 30 to 45 min distributed over the day. They will be recommended to train using the same physical exercises for at least 3 weeks with three sessions per week and a day of rest between each session. Cognitive training instructors will encourage participants to practice the cognitive exercises at least 3 times per week for 15 min each time. Participants will be made aware that during an ideal training session, activity should be perceived as of moderate difficulty. Instructions on physical and cognitive activities will be provided by a different instructor, the same for both programmes. Instructors will specifically be asked to present and explain the two programmes in similar ways.

##### StayFitLonger training programme

**Fig. 1:**
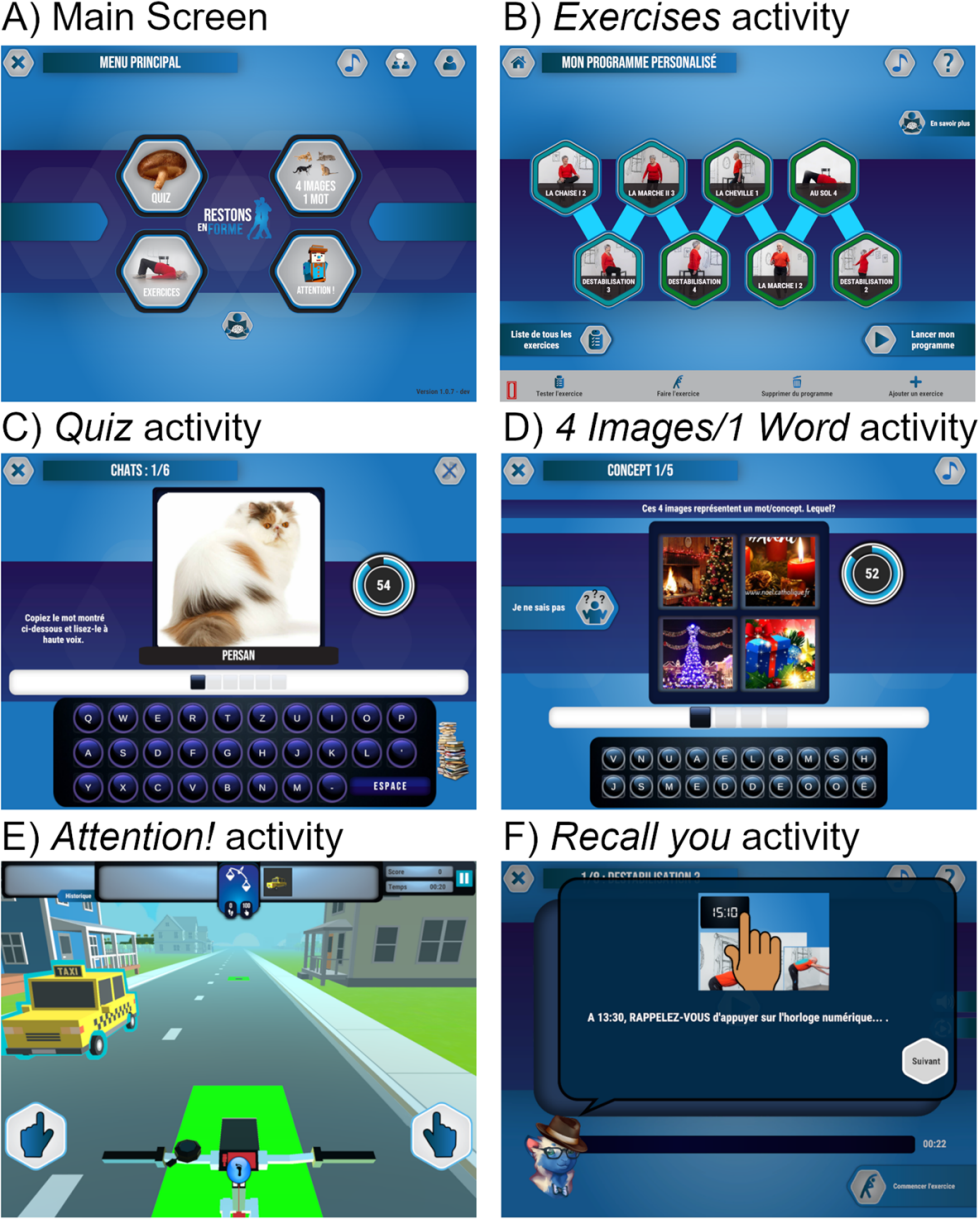
Illustration of the different activities of the StayFitLonger training programme

The StayFitLonger programme will be accessible through the application *RestonsEnForme,* which will be available on a tablet (Galaxy Tab S2, Samsung) that will be provided to each participant. When launched, the main screen of the application provides access to different physical and cognitive activities (Fig. 1a) as well as other features listed below.

The physical exercise activity (*Exercises*) will be based on the T&E home-based programme using the concept of self-efficacy and empowerment [39]. Participants will be invited to create a personalised 8-exercise programme (Fig. 1b). Those will be selected from 50 available exercises, which vary as function of themes (e.g., on a chair, with a pillow) and difficulty level (e.g., different body position or workload). Participants will try the exercises before including them in their programme. Exercices will only be included if judged as not too difficult by the participant. More details on the T&E programme can be found in [39]. During the intervention, participants will be allowed to add new exercises to their 8-exercise programme after a period of at least 3 weeks to introduce variety and increase challenge.

There will be four cognitive training activities which target problem solving, semantic memory, prospective memory, and divided attention. The *Quiz* activity will teach different strategies [49, 50] to learn new vocabulary and semantic repertoires (e.g., mushrooms, trees, flowers, dogs, etc.; Fig. 1c). Participants will choose first a repertoire of interest and will be asked to perform word-image associations related to the repertoire. Based on their level of proficiency in the selected repertoire, participants will then be offered different learning techniques: *completing* (relying on cues for help) or *copying/completing* (copying the word while using pure errorless learning and then completing while using encoding cues). Participants will continue to explore the repertoire through practice using an optimal number of cues to obtain the best performance while limiting the production of errors. This practice will be completed once participants reach at least 60% of correct responses without cues. Then, participants will be invited to a final evaluation without any help. Feedback will be provided with the option of continuing training using the repertoire or choosing another one. The *4 Images/1 Word* activity will train cognitive flexibility (Fig. 1d). Participants will be shown four images that are associated with an overarching concept and will be asked to find and write down the associated concept. Two types of cues will be provided to help them solve the task: number of letters in the target and some of the target letters mixed with distractors. The *Attention!* activity will train participants to vary their attentional priority in dual-tasks [40] while exploring a city on a two-wheel vehicle (Fig. 1e). The dual-task will involve detecting different targets in the environment (i.e., people, 4-wheel vehicles or buildings) by pressing a button on the screen (task A), and at the same time detecting sewer covers with foot taping (task B). The foot response will be recorded by a motion sensor attached to the waist or shoe. The activity will comprise 30 levels with a progressive increase in the degree of difficulty. Difficulty will be increased by manipulating the number of targets, the number of distractors and the speed of the vehicle driven by the player (i.e., bicycle, scooter, motorcycle) and by introducing a response contingency condition (if/then). Participants will complete first each task (detection of targets in the environment and detection of sewer covers) in focused attention. They will then be asked to combine the tasks with different priority levels during a series of trials: one trial in which they will devote 80% of their attention to task A and 20% of attention to task B, one with 20% on task A and 80% on task B, and one with 50% of their attention on each task. Each priority trial will last about 1 min and will be repeated twice in random order. The *Recall you* activity will be embedded into the physical exercises to train prospective memory [51] (Fig. 1f). On every 3 to 4 sessions, the *Exercises* activity will start with an instruction asking participants to complete a casual task (e.g., to get and drink a glass of water or to open a window, etc.) after a certain amount of time in the physical training. A timer will appear on the top left corner so that participants can track time while doing their exercises. For safety reasons, participants will be instructed to complete the exercise they are engaged in before performing the cognitive task.

**Fig. 2:**
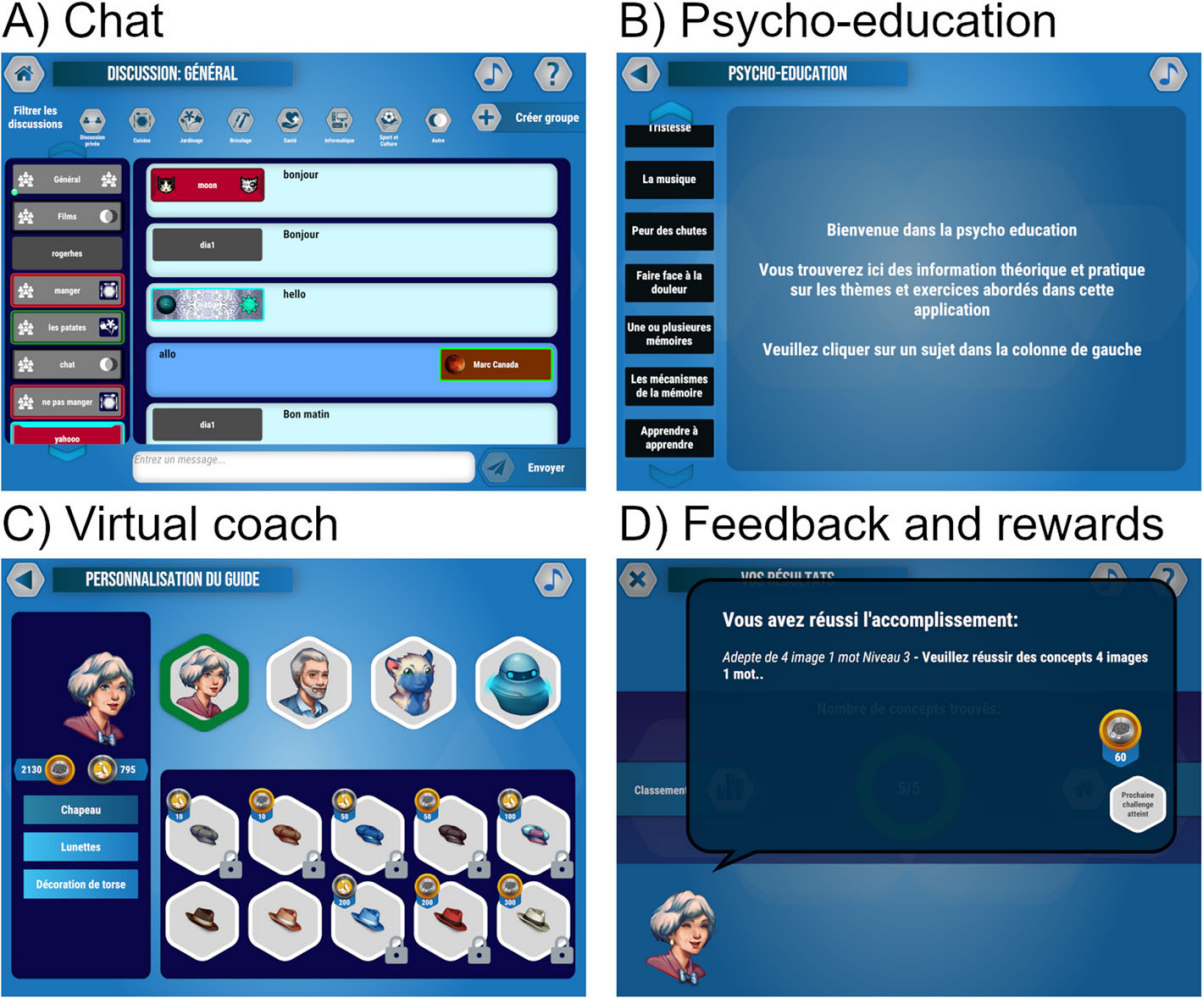
Illustration of the unique features of the StayFitLonger training programme

In addition to the physical and cognitive activities, the StayFitLonger programme will include the following features:

A *Chat room* will provide a venue for participants to share views about topics of interest and tips for common real-life problems (Fig. 2a). Pre-established themes will be available (e.g., cooking, gardening, handiwork, etc.) and participants will have the opportunity to enrich this setting and create their own themes. When entering the chat room, a moderator message will inform participants to be respectful while chatting and to avoid revealing sensitive information (e.g., address, name, credit card information).

*Creation of material*. Participants will be invited to create material for the *4 Images/1 Word* and *Quiz* activities. Once validated by the research team through a moderation platform, the material will be shared with all participants who will have the opportunity to use it for their own training and to rate the material created by other participants. This feature has been implemented to foster social interactions across participants.

*Psychoeducation*. From the application homepage, participants will have access to psychoeducational content (Fig. 2b) on different topics related to physical, psychological and cognitive health. Twenty-two topics will be available (e.g., divided attention improvement; stress regulation; fatigue management, etc.).

*Virtual coach*. A customisable virtual coach using verbal (but written) and non-verbal communication (Fig. 2c, d) will guide participants along the proposed exercises by giving them instructions, reminding them to practice a variety of available activities repeatedly, providing appropriate and timely feedback (through congruent facial expressions) on participant’s performances (e.g., encouraging messages) and rewarding assiduity, perseverance and performance with achievements and virtual credits (“physio-coins” and “cogni-coins”). Some achievements will unlock new icons, backgrounds and frames to modify the user interface, and by spending the coins obtained, it will be possible to get additional icons, background, frames and equipment to customise the virtual coach appearance (e.g., hat, glasses, etc.). These different functions of the virtual coach have been implemented to improve adherence by helping participants through a direct interlocutor (rather than neutral messages) and to keep them motivated [43].

##### Active control training programme

**Fig. 3:**
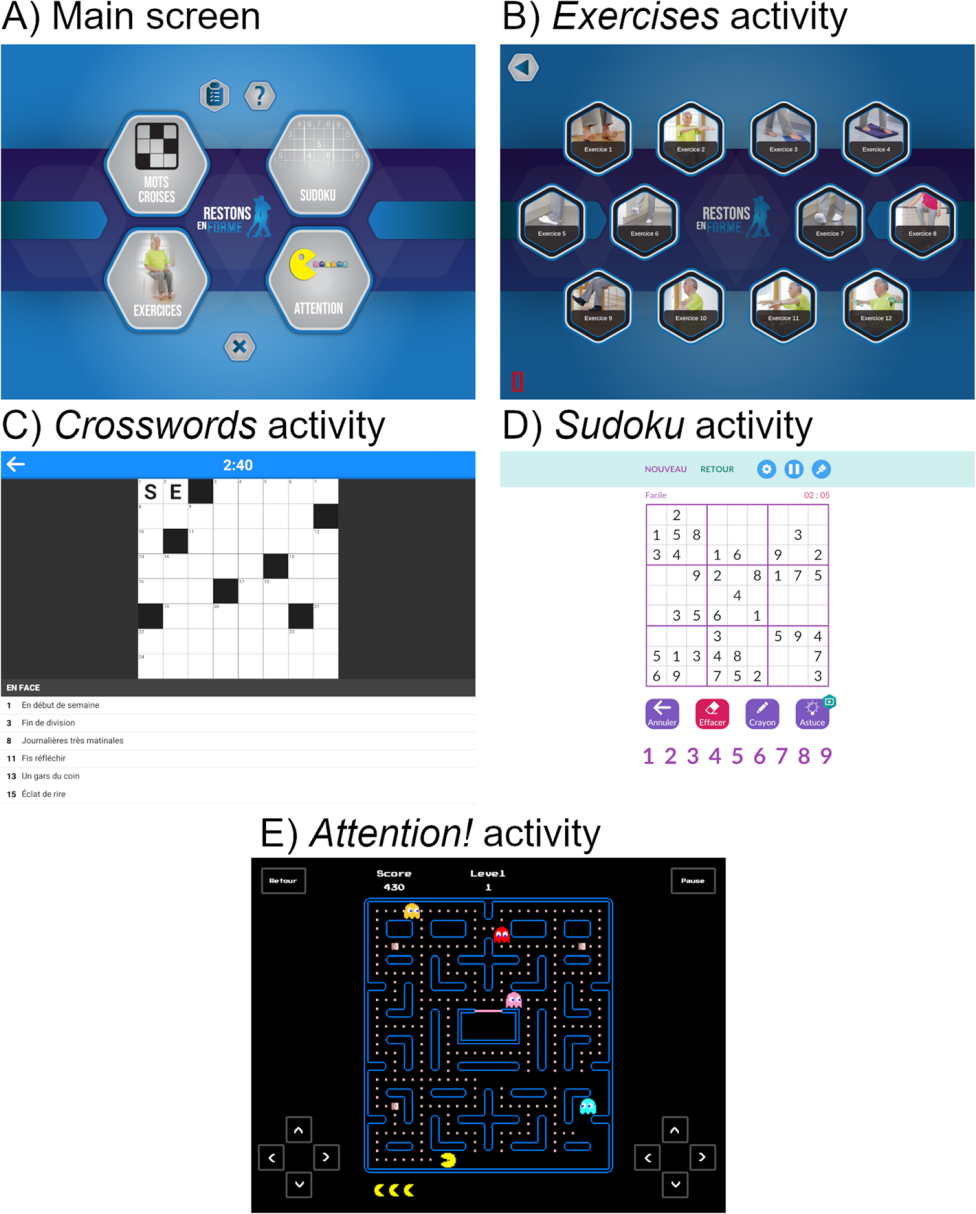
Illustration of the different activities of the active control training programme

The active control programme will be similar in structure and layout to the StayFitLonger programme (Fig. 3a) and will include physical and cognitive exercises.

The physical exercise activity (*Exercises*) will be a computerised version of Helsana’s physical training programme (Fig. 3b). Helsana, a Swiss health insurance company, offers this programme in a booklet. The computerised version will include advice and tips to stay physically active (e.g., to go shopping by foot) and 12 exercises to train upper and lower extremity strength, mobility and balance. It will also provide information about which exercises to choose, the training frequency and precautionary measures to follow. This programme has been judged close to “standard care”, as it is similar to a large range of programmes and recommendations available to the general public. It will differ from the *Exercise* activity available on the StayFitLonger programme, as it only contains a limited number of exercises and does not benefit from interactive content (e.g., videos of exercises), self-management, personalization features, and rewards from the virtual coach.

The four cognitive activities provided in the active control programme will be commercially available leisure activities that do not target specific cognitive processes and do not teach cognitive strategies [52–56]. The *Crosswords* activity will include 219 puzzles with five different sizes (Fig. 3c). The *Sudoku* activity will include around 5000 puzzles with four levels of difficulty (Fig. 3d). The *Attention!* activity will be a maze arcade game inspired from Pac-Man in which participants eat dots in a maze while trying to avoid coloured ghosts (Fig. 3e). The *Countdown* activity will be embedded into the *Exercises* activity and triggered randomly every 3 to 4 days. It will require that participants count backward from 100 to 1 or recite the alphabet from Z to A while doing their exercise.

There will be no chat room, psycho-educational content or virtual coach included in the active control training programme.

##### Supervision during the intervention

Participants will receive a phone call and a home visit on week four and on week eight. Then, they will receive two phone calls (one from the physical activity instructor and one from the cognitive activity instructor) every four weeks. These will serve to identify and help participants to resolve difficulties with the programme, devices or exercises, and to obtain information about their health.

#### Outcome variables

##### Primary outcome

The primary outcome will be the performance on the TUG test [57]. In this test, the person will be sitting on a chair and will be asked to stand-up, walk three meters, turn around, walk back to the chair, and sit down. Time will be measured from the moment the person stands up until s/he sits down. Participants will perform the TUG twice providing two measures that will be averaged.

##### Secondary outcomes

Measures in four domains will be used as secondary outcomes (Table 2).

*Physical domain*. 1) Walking speed will be measured over a 20-m distance. Participants will be instructed to walk as quickly as possible without running and in a safe manner. Time will be measured in seconds using a smartwatch. The task will be carried out twice, and measures will be averaged. 2) Lower extremity strength will be measured with the Five Time Sit to Stand Test (FTSST) [58]. Participants will sit on a chair with arms folded across their chest and will be asked to stand up and sit as quickly as possible five times while keeping their arms folded. The task will be administered twice, and the two measures averaged. 3) In the Four Stage Balance Test (FSBT) [59], participants will be asked to perform four progressively more challenging positions and to hold each of them as long as they can for a maximum of 10 s (parallel, semi-tandem and tandem positions) or as long as possible (one-leg stance position). The test will be stopped if a participant fails at holding a given position. 4) A smartwatch (Huawei Watch 2) connected to two motion Physilog®5 sensors worn by participants will be used during the TUG and the 20-m Walking task to collect additional specific gait movement parameters (Table 2). These sensors are a standalone 7 degree-of-freedom MEMS inertial measurement unit with wireless synchronisation, including 3D accelerometer, 3D gyroscope, and a barometric pressure sensor. The system is non-invasive, as sensors will be directly strapped on right and left shoe/foot.

*Cognitive domain.* 1) Global cognition will be measured with the ZAVEN composite score [60, 61] computed by averaging z-scores from the following tests: delayed free recall of the California Verbal Learning Test (CVLT); delayed recall of the WMS-IV logical memory subtest [62]; number of correct symbols reported in the WAIS-IV digit symbol substitution test (DSST) [63]; and letter fluency of the verbal fluency task [64]. 2) An executive composite score will be computed by averaging z-scores from the following tests: letter fluency of the verbal fluency task; time to complete the Trail Making Test part B-A (TMT) [65]; interference index of the Victoria Stroop Test (VST) [66]; number of total visual and auditory omissions of the divided attention subtest (Test of Attention Performance 2.3.1; TAP [67];). 3) A memory composite score will be obtained by averaging z-scores from the delayed free recall score of the CVLT [68, 69] and the delayed recall of the logical memory task. 4) A processing speed composite score will be obtained by averaging z-scores from the following tests: time to complete the TMT part A; number of correct symbols reported in the DSST; time to complete the “naming condition” of the VST [70]. 5) Divided attention will be measured with a customized computerised task performed on a tablet [40]. Participants will be asked to deliver newspapers by pressing on a screen button while on a bicycle that moves forward automatically. At the same time, they will have to follow the road traffic regulation to ensure their safety (e.g., stopping when traffic lights go from green to red and avoiding animals crossing the road). The tasks will involve different distractors to vary participants’ attentional demand. Participants will be made aware that they should prioritize their safety as they would in real life. Each task will be done first in focused attention and then both tasks will be combined and performed using three levels of speed. The number of delivered items, reaction time, and errors will be recorded. 6) Prospective memory will be measured with two subtests of the Rivermead Behavioural Memory Test (RBMT-3) [71]. In the “belonging” subtest, participants will be instructed to remember asking for two personal belongings at the end of the session. In the “appointment” subtest, participants will be asked to remember asking two questions when an alarm rings 25 min later. 7) Concept elaboration will be assessed with the TAP flexibility sub-test, a “set shifting” computerised task [67] and the WAIS-IV Similarities subtest [63]. In the Similarities subtest, participants will be presented with pairs of words (e.g.: apple and peach) and will be asked how the two words are alike.

*Affective domain*. Mood will be assessed using the Hospital Anxiety and Depression Scale (HADS) [72]. Fear of falling will be measured with the Falls Efficacy Scale-International (FES-I) [73].

*Psychosocial domain.* Quality of life will be assessed with the 35-item Older People Quality of Life questionnaire (OPQOL 35) [74]. Cognition in everyday life will be measured with the self-reported Cognitive Function Instrument (CFI) [75] and Everyday Cognition scale (E-Cog) [76]. The CFI will include 14 questions to measure subjective concerns regarding cognition and activities of daily living over the last year. The E-Cog will measure how cognitive functions in different domains (everyday memory, language, visuospatial abilities, planning, organisation, divided attention) impact activities of daily living compared to 10 years ago. Participant’s expectation toward the efficacy of the training programme will be assessed with an ad-hoc 17-item questionnaire.

#### Statistical analyses

##### Sample size calculation

Given that our secondary analyses will stratify participants into two categories (robust and pre-frail), we determined our sample size to ensure that we have the capacity to test the hypotheses related to this stratification. This was done with a Marker Stratified Design using the following plan: marker-by-treatment interaction using separate test (see: http://www.bigted.org/NonAdaptiveDesigns/MarkerStratifiedDesigns.html). For pre-frail participants, it was estimated that 16 participants per group (StayFitLonger vs. active control) would be required to detect a significant difference of 3.22 s in the TUG test using a two-sided t-test (alpha = 0.05) based on the T&E pilot study. As data might not be normally distributed, a non-parametric test was required resulting in a sample size of about 18 participants per group. Considering a dropout rate of about 25% based on prior studies, a sample size of 24.5 pre-frail participants should be enrolled for each group. For robust older adults, a sample size of 23.5 participants per group would allow to detect a difference of 0.82 s on the TUG test using a two-sided t-test (alpha = 0.05) with a power of 80% based on the study by Uemura et al. [77]. By accounting for the non-normality of data (using a non-parametric test) and the dropout rate, we targeted recruiting 36 robust participants per group. Thus, a total sample of 122 participants was determined as sufficient to have the appropriate power based on sample size calculation. To have a balanced distribution in the three countries, the total N targeted for recruitment was set at 128 participants.

##### Analysis of efficacy on primary and secondary outcomes

All statistical tests will be two-tailed and a *p* value < .05 will indicate statistical significance. Effect sizes will also be assessed. Standard descriptive statistics will be provided with means and standard deviation for demographics and baseline characteristics. Group comparisons will be made using t-tests for continuous variables and chi-square analyses for discrete variables.

The primary efficacy analysis will be done with a modified intention-to treat (mITT) approach. All participants will be included in the analyses and the characteristics of those who withdrew will be analysed, as well as the causes leading them to leave the study. A linear mixed model will be used to analyse the data, as it handles correlated data and unbalanced designs and are robust against missing values. The fixed effects will be Intervention (StayFitLonger vs. active control), Time (T0, T1) and their interaction. If the StayFitLonger training is more beneficial than the active control training, a significant interaction will be expected. In such case, the presence of a significant difference between T0 and T1 in each group will be evaluated, as well as group difference on change scores at post-training using the pre-training and control group as reference points. The same analysis will be used with primary and secondary outcomes. To examine the effect of frailty status on efficacy, participants will be stratified into robust and pre-frail seniors and data will be analyzed separately in these two populations using the same method described above.

##### Analysis of moderators

Age, sex, education and score on MoCA, four variables considered as time-invariant for the duration of the study, will be assessed as potential moderators of the impact of training on primary and secondary outcomes. Prior to their use, we will verify that they are independent from each other with a chi-squared test or correlations.

### Adherence sub-study

#### Design

The adherence sub-study will be a pragmatic quasi-experimental study including all participants from the Swiss and Canadian sites (about 96). Following the 26-week efficacy trial, participants in the experimental group will be asked to continue to use the StayFitLonger programme with no supervision and will be invited to a refresher course to answer questions and discuss potential issues that occurred during the RCT. Participants in the control group will cross-over to use the experimental programme for 22 weeks (Table 1).

#### Variables

*Adherence.* For the entire duration of the study (between T0 and T1, and T1 and T2), three measures of adherence in relation to the device usage will be recorded directly from the application (Table 3): dose measured by the time (min) spent on the programme per week; volume corresponding to the total number of repetitions performed per week within each activity (e.g., 3 quizzes completed while using the Quiz activity); and frequency corresponding to the number of sessions per week. These will be recorded separately for the physical and cognitive activities. Adherence will be calculated for each individual by plotting weekly data over the entire training period.

**Table 3:** List of variables measured during the adherence sub-study

Adherence sub-study variables		Timepoints		
***Domain***	***Variable***	T0	T1	T2
Adherence	Dose variable: total time of training for each activity	During training		
	Volume variable: number of times each activity is carried out			
	Frequency variable: number of training sessions of at least 30 min performed per month			
User experience	Ad-hoc questionnaire exploring the quality of the introductory course	X		
	AttrakDiff 2 scale		X	X
Acceptability	Ad-hoc questionnaire to obtain ratings on different components (enjoyment, appropriateness, safety, self-evaluation)		X	X
Usability	Ad-hoc questionnaire exploring the impact of unique features of the programme (virtual coach, social interactions, preference, gamification, educative content, self-management, usability)			X

*User experience.* The AttrakDiff 2 scale [78] will evaluate user experience (Table 3) with a 28-item questionnaire given at T1 and T2. It will measure attractiveness, pragmatic quality and hedonic qualities (stimulation and identity) of the application. Pragmatic quality corresponds to usability for instance efficiency, effectiveness and learnability. Hedonic qualities refer to the programme’s originality and beauty. In addition, a 9-item questionnaire will be given at T0 to ask participants’ knowledge regarding the effects of cognitive and physical interventions and the quality of the introductory courses.

*Acceptability.* A 9-item ad-hoc questionnaire will be used at T1 and T2 to measure acceptability (Table 3) that is, the participants’ feeling toward the programme (enjoyment, safety, efficacy, motivation to use other programmes) and its appropriateness (for older adults, to improve physical and cognitive health and to maintain a social circle).

*Usability.* A 16-item ad-hoc questionnaire will be used at T2 to measure usability (Table 3). Participants will be asked to rate the virtual coach, social interactions, gamification, educative content, and self-management.

#### Statistical analyses

Dose, frequency and volume variables will be analysed with polynomial regression models including linear and non-linear trajectories. This will allow to establish the best fitting model describing the use of the programme over time. Regression analyses will examine whether the cumulative values on adherence variables are predicted by personal characteristics, and the group to which they were assigned. We will also examine adherence dichotomously by classifying participants as a function of whether they maintain or not the recommended dose of the programme over time. Finally, we will evaluate whether change scores on primary and secondary measures of efficacy are correlated with adherence.

Qualitative analyses will be used to characterize usability, acceptability and user experience. In addition, correlations analyses between these parameters and a series of variables (age, sex, education, cognitive profile, participant’s technology and gaming profile) will be performed.

### Quality control and monitoring

Several strategies will be implemented to ensure quality control of the data and intervention. The introductory sessions will be standardised to ensure consistency between sites and between instructors. Assessors will be trained on the tasks with videos and will complete mock testing sessions that will be used to assess adherence to the protocol. Regular controls will be done regarding recruitment and assessment by site coordinators. Any modification to the protocol will be shared with the investigators and among sites and reported to the ethics committee. Internal audits will be conducted to ensure the proper conduct of the study and to certify that it complies with the protocol. Shortly after the beginning of the study and again once the study is completed, data from five participants will be selected randomly and verified by a researcher not involved in data collection. This person will ensure that informed consents have been signed, that eligibility criteria have been respected, and that all the tests relative to the assessments have been properly completed and original documents scanned and uploaded in REDCap.

### Potential harms

Falls are one of the adverse events that could occur during the study. Participants will be asked to report the occurrence of a fall and its severity in the last year for T0, and in the T0-T1 and T1-T2 periods [79]. In addition, participants will be asked to report and discuss any potential adverse event during home visits and phone calls. These will be classified according to the typology developed in [80]: falls that require medical attention; exacerbation of a pre-existing illness; increase in the frequency or intensity of a pre-existing episodic event or condition; condition detected or diagnosed after the intervention, even though it may have been present prior to the start of the study; continuous persistent disease or symptoms present at T0 that worsened following the start of the study. All adverse events will be assessed for severity, expectedness and causality and will be recorded and closely monitored until resolution or stabilisation or until it is shown that the study intervention was not the cause.

### Access to data

All site coordinators and principal investigators will be given access to the cleaned data sets. They will have direct access to their own site’s data sets, and will have access to other sites data by request. To ensure confidentiality, data dispersed to research team members will be blinded of any identifying participant information.

### Dissemination of study results

Study results will be published in international journals with peer-reviewed committees. They will also be presented to the research community in national and international conferences and to the public through lay audience talks and press releases. Interim analyses will be conducted once a site has completed the RCT as required by one of the funding organization (the Active and Assisted Living Programme).

## Trial status

Protocol CER-VD 2018–01898 version number 2, December 2018. Recruitment began in January 2019 in Switzerland and Canada and in January 2020 in Belgium. Recruitment was on hold between March and July 2020 in Belgium due to COVID-19. As of August 7, 2020, recruitment has been completed in Canada and Switzerland and has resumed in Belgium. Date of recruitment completion is anticipated to be October 2020.

## Discussion

This study will measure the effect of the StayFitLonger programme, a computerised home-based training, which combines physical and cognitive activities and includes elements to favour social life as well as feedback and instructions from a virtual coach to enhance motivation and adherence. The overarching objectives of the study are: 1) to provide scientific evidence that such a programme can promote physical and cognitive health while staying at home; 2) to examine the level and determinants of adherence to a home-based computerised programme as well as assessing participants’ perception of the programme and its functionalities.

The StayFitLonger programme includes several innovative features. First, while most physical activity interventions designed for older adults rely on aerobic training, the StayFitLonger programme focuses on balance and strength and was originally designed for older adults at risk of falling. Encouraging pilot results (T&E study, unpublished data) indicated an improvement in balance after 6 months compared to home-based exercise programme. Therefore, we believe that this approach could be beneficial to older adults at risk of frailty and that it could improve strength and reduce falls. The inclusion of pre-frail and robust participants will offer the opportunity to assess the impact and relevance of the programme for older adults with a range of physical capabilities. If necessary, there will be an opportunity to adjust the content in terms of difficulty so that it can broaden the targeted population of the programme for future use or studies. Another innovation is the use of motion sensors during physical exercises and as an outcome, which will provide precise and objective measurements on mobility for a better characterisation of how participants complete the exercises and on physical improvements.

Although the StayFitLonger intervention focuses on cognitive and physical training, it includes complementary approaches that could potentiate its effect, in particular the possibility for participants to interact with other players, promoting an active social life [42], and the inclusion of psychoeducational content. In addition, the virtual coach will provide some elements of feedback and reward, which is expected to increase motivation and adherence [43]. This is innovative, as participants in home-based training benefit from limited training assistance and coaching and this negatively impacts use over the long-term.

One important aspect of the study is to provide a direct measure of adherence, and to follow participants beyond the RCT, which will provide adherence data under unsupervised and more pragmatic conditions. To our knowledge there is no consensus on a method to assess adherence and therefore many different approaches are used in the literature with varying limitations [81]. Precise measurement of adherence to a home-based exercise programme is limited by the need to rely on self-reported measures. However, the use of a computer programme makes it possible to measure the use of the tablet and different exercises to the minute. This will provide a rich set of accurate data on dose, volume and frequency of training.

While the efficacy of a computerised training programme is essential, one prerequisite to its use by the population is that the programme is easy to use and matches the needs of older adults.

The StayFitLonger study will provide critical information on usability, acceptability and user experience, which are inter-related concepts providing insights on the potential long-term use of a technology [36, 37]. Usability refers to the ease of use of a technology and is characterized as a person’s perception of its efficacy, efficiency and satisfaction [82]. Acceptability is an a priori willingness to use a tool, while acceptance is an a posteriori pragmatic evaluation of a tool after its use [83]. They are known to be influenced by usability but also by other factors, such as perceived usefulness by the user and others, and this is particularly true among seniors [82]. User experience, which refers to a person’s perceptions and responses that result from the technology use and/or anticipated use is also dependant on usability and has an impact on acceptability [84]. Results obtained on these parameters are important to determine the factors that influence future use, but they can also help improving the application or designing new solutions to provide the best experience for end users.

The design includes an active control training as a comparator that mirrors the structure of the StayFitLonger training. Using an active control condition will allow us to attribute the improvement to the particular content and format of the StayFitLonger programme. Relying on an active control condition as a comparator is a methodological strength compared to other studies that used a no-contact or wait-list control condition. However, this comes with additional challenges in terms of design and power. One of these challenges is that we need to ensure that participants in each group have the same expectation regarding the capacity of their assigned training to yield improvement [48]. To control for this effect, we included an expectation scale and will assess whether it differs among groups and whether expectations are related to pre-post changes.

We are aware of the potential limitations associated with this study. First, the StayFitLonger study spans over a full year and it is hard to predict whether participants will commit for such an extended period of time. Note, however, that we rely on a two-part design, with the RCT portion only lasting 26 weeks, and that attrition will be examined separately for the two portions. Second, while the use of feedback and rewards in the programme was meant to boost motivation, the frequency at which they appear has been set as a fixed parameter to avoid a bias between participants. Hence, this aspect was not personalized. In order to take into account that some participants may dislike receiving regular feedback, they will be able to deactivate this feature during the adherence sub-study. Adherence will be measured from the tablet application, but it is possible that some participants, intentionally or not, start an activity and let the application run in the background while they are actually not doing the activity. To counteract this possibility, the StayFitLonger programme includes a function that stops the application after 10 min of inactivity. However, it has not been implemented in the active control programme. This will be monitored as carefully as possible during data analysis to assess possible outliers in the time of use data of the active control group.

In conclusion, the StayFitLonger study will examine the efficacy, adherence and perception of a home-based computerised multi-modal training programme in robust and pre-frail older adults. Positive results on the StayFitLonger study will pave the way to further development and commercialisation of a scientifically grounded and empirically validated application which will improve the physical and cognitive health associated with independent life at home.

## Data Availability

Not applicable.

## Abbreviations

4-IADL: 4-Instrumental Activities of Daily Living
AD: Alzheimer’s Disease
ANOVA: ANalysis Of VAriance
CFI: Cognitive Function Instrument
CHUV: Centre Leenaards de la mémoire – Centre hospitalier universitaire Vaudois
CIUSSS: Centre intégré universitaire de santé et de services sociaux
CVLT: California Verbal Learning Test
DSST: Digit Symbol Substitution Test
E-Cog: Everyday Cognition Scale
FES-I: Falls Efficacy Scale-International
FSBT: Four Stage Balance Test
FTSST: Five Time Sit to Stand Test
HADS: Hospital Anxiety and Depression Scale
HES-SO: Haute École Spécialisée de Suisse Occidentale
IUGM: Institut universitaire de gériatrie de Montréal
MCI: Mild Cognitive Impairment
mITT: Modified Intention-To Treat
OPQOL 35: Older People Quality of Life questionnaire
RBMT-3: Rivermead Behavioural Memory Test – Third edition
RCT: Randomised Control Trial
TAP: Test of Attention Performance 2.3.1
T&E: Test-and-Exercise home-based programme
TMT: Trail Making Test
TUG: Timed Up & Go
VST: Victoria Stroop Test
WMS-IV: 4th version of the Wechsler Memory Scale
WAIS-IV: 4th version of the Wechsler Adult Intelligence Scale
ZAVEN: Z-scores of Attention, Verbal fluency, and Episodic memory for Nondemented older adults
